# Heterogeneous Reconstruction of Tracks and Primary Vertices With the CMS Pixel Tracker

**DOI:** 10.3389/fdata.2020.601728

**Published:** 2020-12-21

**Authors:** A. Bocci, V. Innocente, M. Kortelainen, F. Pantaleo, M. Rovere

**Affiliations:** ^1^ CERN, European Organization for Nuclear Research, Meyrin, Switzerland; ^2^ Fermi National Accelerator Laboratory, Batavia, IL, United States

**Keywords:** GPU, CMS, heterogeneous computing, patatrack, particle track reconstruction, vertex reconstruction

## Abstract

The High-Luminosity upgrade of the Large Hadron Collider (LHC) will see the accelerator reach an instantaneous luminosity of 7 × 10^34^ cm^−2^ s^−1^ with an average pileup of 200 proton-proton collisions. These conditions will pose an unprecedented challenge to the online and offline reconstruction software developed by the experiments. The computational complexity will exceed by far the expected increase in processing power for conventional CPUs, demanding an alternative approach. Industry and High-Performance Computing (HPC) centers are successfully using heterogeneous computing platforms to achieve higher throughput and better energy efficiency by matching each job to the most appropriate architecture. In this paper we will describe the results of a heterogeneous implementation of pixel tracks and vertices reconstruction chain on Graphics Processing Units (GPUs). The framework has been designed and developed to be integrated in the CMS reconstruction software, CMSSW. The speed up achieved by leveraging GPUs allows for more complex algorithms to be executed, obtaining better physics output and a higher throughput.

## 1. Introduction

The High-Luminosity upgrade of the LHC (Apollinari et al., 2017) will pose unprecedented challenges to the reconstruction software used by the experiments due to the increase both in instantaneous luminosity and readout rate. In particular, the CMS experiment at CERN (CMS Collaboration, 2008) has been designed with a two-levels trigger system: the Level 1 Trigger, implemented on custom-designed electronics, and the High Level Trigger (HLT), a streamlined version of the CMS offline reconstruction software running on a computer farm. A software trigger system requires a trade-off between the complexity of the algorithms running on the available computing resources, the sustainable output rate, and the selection efficiency.

When the HL-LHC will be operational, it will reach a luminosity of 7 × 10^34^ cm^−2^s^−1^ with an average pileup of 200 proton-proton collisions. To fully exploit the higher luminosity, the CMS experiment will increase the full readout rate from 100 to 750 kHz (CMS Collaboration, 2020). The higher luminosity, pileup and input rate present an exceptional challenge to the HLT, that will require a processing power larger than today by more than an order of magnitude.

This exceeds by far the expected increase in processing power for conventional CPUs, demanding alternative solutions.

A promising approach to mitigate this problem is represented by *heterogeneous computing*. Heterogeneous computing systems gain performance and energy efficiency not by merely increasing the number of the same-kind processors, but by employing different co-processors specifically designed to handle specific tasks in parallel. Industry and High-Performance Computing centers (HPC) are successfully exploiting heterogeneous computing platforms to achieve higher throughput and better energy efficiency by matching each job to the most appropriate architecture.

In order to investigate the feasibility of a heterogeneous approach in a typical High Energy Physics experiment, the authors developed a novel pixel tracks and vertices reconstruction chain within the official CMS reconstruction software CMSSW (Jones et al., 2006), using the CUDA parallel computing platform (Nickolls et al., 2008). The input to this chain is represented by RAW data coming out directly from the detector’s front-end electronics, while the output is represented by legacy pixel tracks and vertices that could be transparently re-used by other components of the CMS reconstruction.

The results shown in this article are based on the Open Data and the data formats released (CMS Collaboration, 2018).

The development of a heterogeneous reconstruction faces several fundamental challenges:

- the adoption of a different programming paradigm;
- the experimental reconstruction framework and its scheduling must accommodate for heterogeneous processing;
- the heterogeneous algorithms should achieve the same or better physics performance and processing throughput as their CPU siblings;
- it must be possible to run and validate on conventional machines, without any dedicated resources.

This article is organized as follows: Section 2 will describe the CMS heterogeneous framework, Section 3 will discuss the algorithms developed in the Patatrack pixel track and vertex reconstruction workflow, Section 4 will describe the physics results and computational performance and compare them to the CMS pixel track reconstruction used at the HLT for data taking in 2018, while Section 5 will contain our conclusions.

## 2. Software Framework

### 2.1. CMSSW

The backbone of the CMS data processing software, CMSSW, is a rather generic framework that processes independent chunks of data (Jones et al., 2006). These chunks of data are called events, and in CMS correspond to one full readout of the detector. Consecutive events with uniform calibration data are grouped into luminosity blocks, that are further grouped into longer runs.

The data are processed by modules that communicate via a C++-type-safe container called event (or luminosity block or run for the larger units). An analyzer can only read data products, while a producer can read and write new data products and a filter can, in addition, decide whether the processing of a given event should be stopped. Data products become immutable (or more precisely, const in the C++11 sense) after being inserted into the event.

During the Long Shutdown 1 and Run 2 of the LHC, the CMSSW framework gained multi-threading capabilities (Jones and Sexton-Kennedy, 2014; Jones et al., 2015; Jones 2017) implemented with the Intel Threading Building Blocks (TBB) software library. The threading model employs task-level parallelism to process concurrently independent modules within the same or different events, multiple events within the same or different luminosity blocks and intervals of validity of the calibration data. Currently the boundary of runs incur barrier-style synchronization point in the processing. A recent extension is the concept of *external worker*, a generic mechanism to allow producers in CMSSW to offload asynchronous work outside of the framework scheduler. More details on the concept of external worker and its interaction with CUDA can be found in (Bocci et al., 2020).

## 3. Patatrack Pixel Track and Vertex Reconstruction Strategy

The reconstruction of the trajectories of charged particles recorded in the silicon pixel and silicon strip detectors is one of the most important components in the interpretation of the detector information of a proton-proton collision. It provides a precise measurement of the momentum of charged particles (muons, electrons and charged hadrons), the identification of interaction points of the proton-proton collision (primary vertex) and decay points of particles with significant lifetimes (secondary vertices).

**FIGURE 1:**
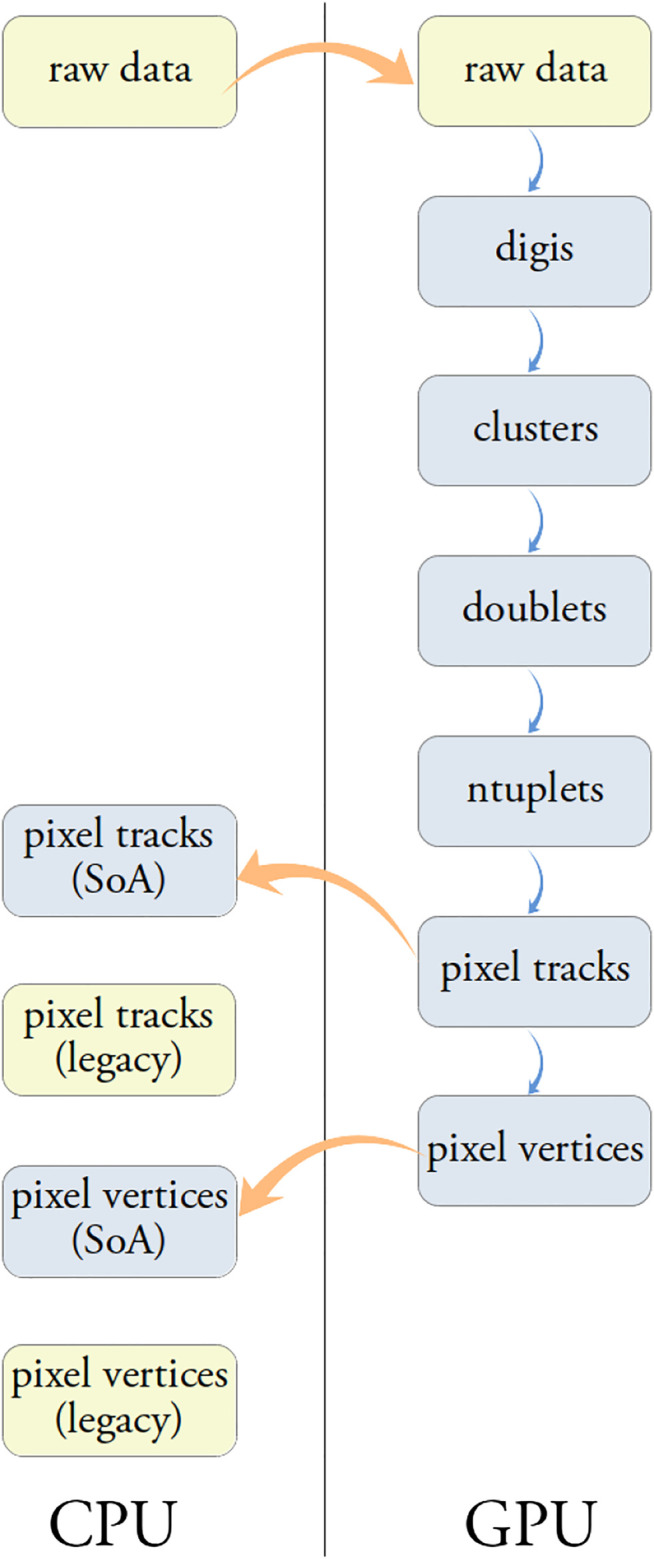
Steps involved in the tracks and vertices reconstruction starting from the pixel “raw data”.

Precise track reconstruction becomes more challenging at higher pileup, as the number of vertices and the number of tracks increase, making the pattern recognition and the classification of hits produced by the same charged particle a harder combinatorial problem. To mitigate the complexity of the problem the authors developed parallel algorithms that can perform the track reconstruction on GPUs, starting from the “raw data” from the CMS Pixel detector, as will be described later in this section. The steps performed during the tracks and vertices reconstruction are illustrated in Figure 1.

The data structures (structure of arrays, SoA) used by the parallel algorithms are optimized for coalesced memory access on the GPU and differ substantially from the ones used by the standard reconstruction in CMS (legacy data formats). The data transfer between CPU and GPU and their transformation between legacy and optimized formats are very time consuming operations. For this reason the authors decided to design the *Patatrack* reconstruction as a fully contained chain of modules that runs on the GPU starting from the “raw data” and produces the final tracks and vertices as output. While a “mixed CPU-GPU workflow” is not supported, for validation purposes the intermediate data products can be transferred from the GPU to the CPU and converted to the corresponding legacy data formats.

### 3.1. Local Reconstruction in the Pixel Detector

**FIGURE 2:**
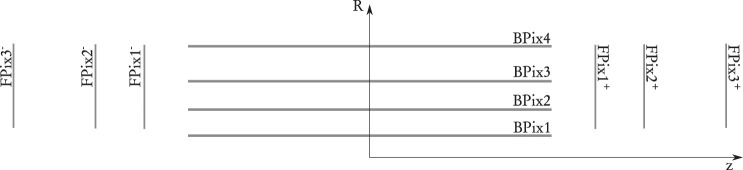
Longitudinal sketch of the pixel detector geometry.

The CMS “Phase 1” Pixel detector (Dominguez et al., 2012), installed in 2017, will serve as the vertex detector until the major “Phase 2” upgrade for the HL-LHC. It consists of 1856 sensors of size 1.6 cm by 6.3 cm each with 66,560 pixels, for a total of 124 million pixels, corresponding to about 2 m^2^ total area. The pixel size is 100µm × 150 μm, the thickness of the sensitive volume is 285 μm. The sensors are arranged in four “barrel” layers and six “endcap” disks, three on each side, to provide four-hit pixel coverage up to a pseudorapidity of |η|<2.5. The CMS pixel detector geometry is sketched in Figure 2. The barrel layers extend for 26.7 cm on each side of the center of the detector. The innermost barrel sensors are located at radius of 2.7 cm from the beam line, and the farthest at 16.4 cm. The forward disks are located between 32 and 48 cm from the center of the detector along the beam line and cover radii between 4.6 and 16 cm. While hermeticity is guaranteed along the azimuthal coordinate thanks to sensor overlaps, gaps exist between sensors along the direction of the beam in the barrel and in the radial direction in the endcaps. A larger gap exists between the barrel and each endcap.

The analog signals generated by charged particles traversing the pixel detectors are digitized by the read-out electronics and packed to minimize the data rate. The first step of the track reconstruction is thus the *local reconstruction*, that reconstructs the information about the individual hits in the detector.

During this phase, the digitized information is unpacked and interpreted to create *digis*: each *digi* represents a single pixel with a charge above the signal-over-noise threshold, and contains information about the collected charge and the local row and column position in the module grid. This process is parallelized on two levels: information coming from different modules is processed in parallel by independent blocks of threads, while each digi within a module is assigned a unique index and is processed by a different thread.

Neighboring digis are grouped together to form *clusters* using an iterative process. Digis within each module are laid out on a two dimensional grid using their row, column and unique index information. Each digi is then assigned to a thread. If two or more adjacent digis are found, the one with the smaller index becomes the *seed* for the others. This procedure is repeated until all the digis have been assigned to a seed and no other changes are possible. The outcome of the clusterization is a cluster index for each digi: a thread is allocated to each seed; a global atomic counter is increased by all threads, returning the unique cluster index for each seed, and thus for each digi.

Finally, the shape of the clusters and the charge of the digis are used to determine the *hit position* and its uncertainty in the coordinates local to the module as described in [(CMS Collaboration, 2014), §3.1].

### 3.2. Building *n*-tuplets

Clusters are linked together to form *n*-tuplets that are later fitted to extract the final track parameters. The *n*-tuplets production proceeds through the following steps:

- creation of doublets
- connection of doublets
- identification of root doublets
- Depth-First Search (DFS) from each root doublet

**FIGURE 3:**
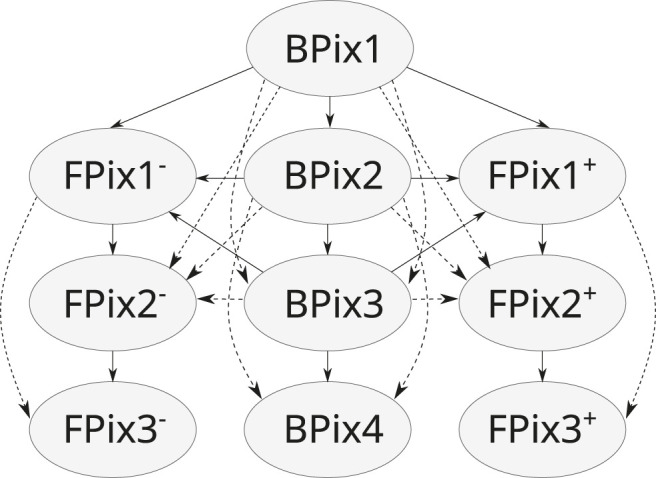
Combinations of pixel layers that can create doublets directly (solid arrow), or by skipping a layer to account for geometrical acceptance (dashed arrow).

The doublets are created by connecting hits belonging to adjacent pairs of pixel detector layers, illustrated by the solid arrows in Figure 3. To account for geometrical and detector inefficiency doublets are also created between chosen pairs of non-adjacent layers, as illustrated by the dashed arrows in Figure 3.

**FIGURE 4:**
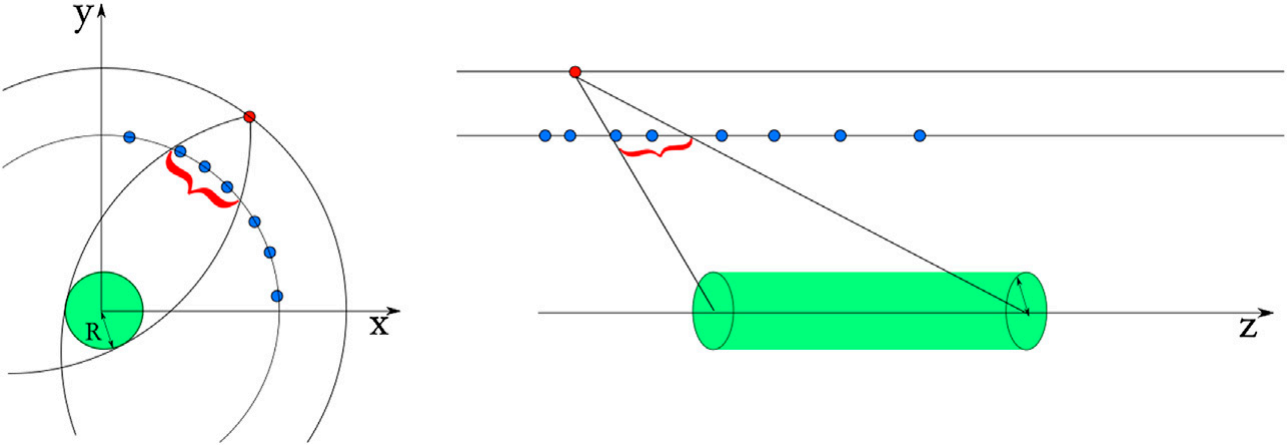
Windows opened in the transverse and longitudinal planes. The outer hit is colored in red, the inner hits in blue (Pantaleo, 2017).

Various selection criteria are applied to reduce the combinatorics. The following criteria have a strong impact on timing and physics performance:

- $p_{\text{T}}^{\text{min}}$: searching for low transverse momentum tracks can be very computationally expensive. Setting a minimum threshold for $p_{\text{T}}$ limits the possible curvature, hence reducing the number of possible combinations of hits.
- $R^{\text{max}}$ and $z^{\text{max}}$: the maximum transverse and longitudinal distance of closest approach with respect to the beam-spot. Tracks produced within a radius of less than 1 mm around the beam-spot are called *prompt tracks.* Searching for *detached tracks* with a larger value of $R^{\text{max}}$ leads to an increase in combinatorics. These “alignment criteria” are illustrated in Figure 4.
- $n_{\text{hits}}$: requiring a high number of hits in the *n*-tuplets leads to a more pure set of tracks and cuts can be loosened, while a lower number of hits produces higher efficiency at the cost of a higher fake rate.

Hits within each layer are arranged in a tiled data-structure along the azimuthal (ϕ) direction for optimal performance. The search for compatible hit pairs is performed in parallel by different threads, each starting from a different outer hit. The pairs of inner and outer hits that satisfy the alignment criteria and have compatible clusters sizes along the *z*-direction form a doublet. The cuts applied during the doublets building are described in Table 1, and their impact on the physics results and reconstruction time are provided in Tables 2, 3.

**TABLE 1:** Description of the cuts applied during the reconstruction of doublets.

Cut	Description
PhiHist	Binned phi window between inner and outer hit using a 128 bin histogram
PhiW	PhiHist + tuned phi window between inner and outer hit
ZW	Window in z for the inner hit
ZIP	Cut on the impact parameter along the beam axis
PT	Cut on the curvature assuming zero transverse impact parameter (TIP), equivalent to a cut on the TIP for high $p_{\text{T}}$ tracks
CSZ	Cut on the cluster size compatibility

**TABLE 2:** Average number of doublets, *n*-tuplets and final tracks per event, as well as the fraction of cells not connected, for each set of doublet reconstruction cuts (described in Table 1), running over a sample of $\text{t}\bar{\text{t}}$ events with an average pileup of 50 and an average of 15,000 hits per event.

Cuts	Doublets	*n*-tuplets	Tracks	Not connected
PhiHist	1,268,193	23,254	1,256	0.966
PhiHist + ZW	866,316	18,301	1,266	0.966
PhiHist + ZW + ZIP	269,410	11,235	1,265	0.926
PhiW + ZW	594,739	13,403	1,212	0.958
PhiW + ZW + ZIP	185,642	8,327	1,214	0.919
PhiW + ZW + ZIP + CSZ	129,307	6,060	1,087	0.915
PhiW + ZW + ZIP + PT	164,567	7,273	1,141	0.921
PhiW + ZW + ZIP + PT + CSZ	115,248	5,270	999	0.918

**TABLE 3:** Time spent in the three components of *n*-tuplets building (as described in the text), as well as in the *Fishbone* and ambiguity resolution algorithms (“clean”), for each set of doublet reconstruction cuts (described in Table 1). It should be noted that using very relaxed cuts requires larger memory buffers on GPU, up to 12 GB, while running with the last four sets requires less than 2 GB of memory.

	Time in μs			
Cuts	Doublets	Connect	DFS	Clean
PhiHist	6,123	15,127	1,690	1,976
PhiHist + ZW	950	6,582	778	538
PhiHist + ZW + ZIP	310	488	354	237
PhiW + ZW	552	2,995	549	377
PhiW + ZW + ZIP	271	265	274	183
PhiW + ZW + ZIP + CSZ	291	187	216	154
PhiW + ZW + ZIP + PT	259	156	246	125
PhiW + ZW + ZIP + PT + CSZ	280	108	192	114

The doublets that share a common hit are tested for compatibility to form a triplet. The compatibility requires that the three hits are aligned in the R−z plane, and that the circumference passing through them intersects the beamspot compatibility region defined by $R^{\text{max}}$. All doublets from all layer pairs are tested in parallel.

All compatible doublets form a direct acyclic graph. All the doublets whose inner hit lies on *BPix1* are marked as root doublets. To reconstruct “outer” triplets, doublets starting on *BPix2* or the two *FPix1* layers and without inner neighbors are also marked as root. Each root doublet is subsequently assigned to a different thread that performs a Depth-First Search (DFS) over the direct acyclic graph starting from it. A DFS is used because one could prefer searching for all the *n*-tuplets up to *n* hits. The advantage of this approach is that the buckets containing triplets and quadruplets are disjoint sets as a triplet could not have been extended further to become a quadruplet (Pantaleo, 2017).

### 3.3. *Fishbone* *n*-tuplets

**FIGURE 5:**
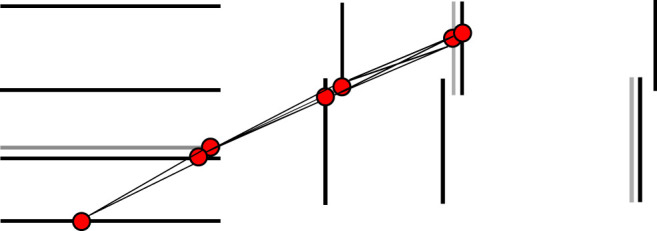
A typical *Fishbone* *n*-tuplets. The shadowed areas indicate partially overlapping modules in the same layer.

Full hit coverage in the instrumented pseudorapidity range is implemented in modern Pixel Detectors via partially overlapping sensitive layers. This, at the same time, mitigates the impact of possible localized hit inefficiencies. With this design, though, requiring at most one hit per layer can lead to several *n*-tuplets corresponding to the same particle. This is particularly relevant in the forward region due to the design of the Pixel Forward Disks that is illustrated in Figure 5: up to four hits in the same layer can be found in localized forward areas. The *Fishbone n*-tuplet solves the ambiguities by merging overlapping doublets. The *Fishbone* mechanism is active while creating the doublets: among all the aligned doublets that share the same outermost hit, only the shortest one is kept. In this way ambiguities are resolved and a single *Fishbone n*-tuplet is created.

Furthermore, among all the tracks that share a hit-doublet only the ones with the largest number of hits are retained.

### 3.4. *n*-tuplet Fit

The “Phase 1” upgraded pixel detector has one more barrel layer and one additional disk at each side with respect to the previous detector. The possibility of using four (or more) hits from distinct layers opens new opportunities for the pixel tracker fitting method. It is possible not only to give a better statistical estimation of the track parameters (${\text{d}}_{z}$, cot(θ), ${\text{d}}_{0}$, $p_{\text{T}}$ and ϕ (Frühwirth et al., 2000) thanks to the additional points, but also to include in the fitting procedure more realistic effects, such as the energy loss and the multiple scattering of the particle due to its interaction with the material of the detector.

The pixel track reconstruction developed by the authors includes a multiple scattering-aware fit: the Broken Line (Blobel, 2006) fit. This follows three main steps:

- a fast pre-fit in the transverse plane gives an estimate of the track momentum, used to compute the multiple scattering contribution,
- a line fit in the s-z plane (Frühwirth et al., 2000),
- a circle fit in the transverse plane.

The ${\text{d}}_{z}$ and cot(θ) track parameters and their covariance matrices are derived from the line fit, while the ${\text{d}}_{0}$, $p_{\text{T}}$ and ϕ, and their covariance matrices from the circle fit. The final track parameters and covariance matrix are computed combining the individual results together.

The fits are performed in parallel over all *n*-tuplets using one thread per *n*-tuplet. The fit implementation uses the Eigen C++ library (Guennebaud et al., 2010) that natively supports CUDA.

### 3.5. Ambiguity Resolution

Tracks that share a hit-doublet are considered “ambiguous” and only the one with the best $\chi^{2}$ is retained. Triplets are considered “ambiguous” if they share one hit: only the one with the smallest transverse impact parameter is retained.

### 3.6. Pixel Vertices

The fitted pixel tracks are subsequently used to form pixel vertices. Vertices are searched as clusters in the *z* coordinate of the point of closest transverse approach of each track with the beam line $\left(z_{0}\right)$. Only tracks with at least four hits and a $p_{\text{T}}$ larger than a configurable threshold (0.5 GeV) are considered. For each track with an error in $z_{0}$ lower than a configurable threshold the local density of close-by tracks is computed. Tracks are considered in the density calculation if they are within a certain $\text{Δ}z_{\text{cut}}$ and if their $\chi^{2}$ compatibility is lower than a configurable $\chi_{\text{threshold}}^{2}$. Tracks with local density greater than 1 are considered as a seed for a vertex. Tracks are then linked to another track that has a higher local density, if the distance between the two tracks is lower than $\text{Δ}z_{\text{cut}}$ and if their $\chi^{2}\leq \chi_{\text{threshold}}^{2}$. All the tracks that are logically linked starting from each seed become part of the same vertex candidate. Each vertex candidate is promoted to be a final vertex if it contains at least 2 tracks.

This algorithm is easily parallelizable and, in one dimension as in this case, requires no iterations. As showed below this algorithm is definitively more efficient and has comparable resolution than the “gap” algorithm used so far at the CMS HLT [(CMS Collaboration, 2014), §6.2].

Each vertex position and error along the beam line are computed from the weighted average of the $z_{0}$ of the contributing tracks. Vertices with a $\chi^{2}$ larger than a given threshold (9 per degree of freedom) are split in two using a *k-mean* algorithm.

Finally the vertices are sorted using the sum of the $p_{\text{T}}^{2}$ of the contributing tracks. The vertex with the largest $\sum^{\text{​}}p_{\text{T}}^{2}$ is labeled as the “primary” vertex i.e. the vertex corresponding to the signal (triggering) event.

## 4. Results

In this section the performance of the Patatrack reconstruction is evaluated and compared to the track reconstruction based on pixel quadruplets that CMS has used for data taking in 2018 (in the following referred to as CMS-2018) (Pantaleo, 2017).

### 4.1. Input Dataset

The performance studies have been performed using 20,000 t$\bar{\text{t}}$ simulated events from CMS Open Data (CMS Collaboration, 2018), with an average of 50 superimposed pileup collisions with a center-of-mass energy $\sqrt{s}=13$ TeV, using detector design conditions.

### 4.2. Physics Performance

The efficiency is defined as the fraction of simulated tracks, $N_{\text{sim}}$, having produced at least three hits in the pixel detector, that have been associated with at least one reconstructed track, $N_{\text{rec}}$.

$$\text{efficiency}=\frac{N_{\text{rec}}}{N_{\text{sim}}}.$$ (4.1)

**FIGURE 6:**
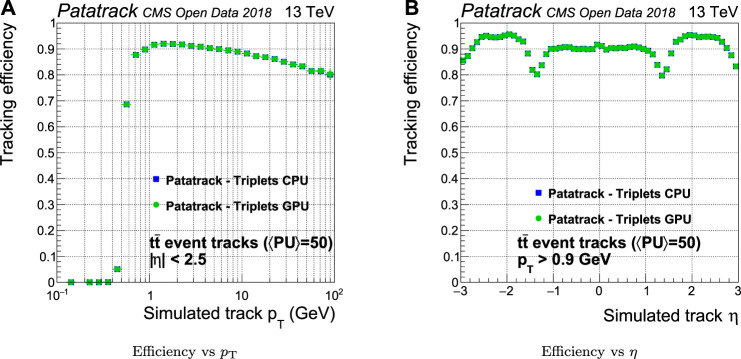
Comparison of the pixel tracks reconstruction efficiency of the CPU and GPU versions of the Patatrack pixel reconstruction for simulated t$\bar{\text{t}}$ events with an average of 50 superimposed pileup collisions.

A reconstructed pixel track is associated with a simulated track if all the hits that it contains come from the same simulated track. The efficiency is computed only for tracks coming from the hard interaction and not for those from the pileup. The CPU and GPU versions of the Patatrack workflow produce the same physics results, as shown in Figure 6. For this reason, there will be no further distinction in the discussion of the physics results between the workflows running on CPU and GPU.

**FIGURE 7:**
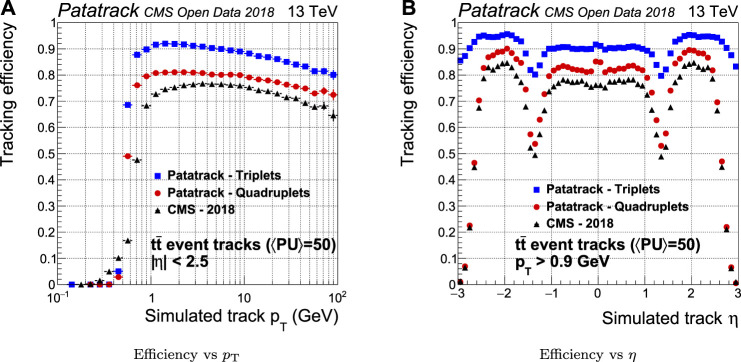
Pixel tracks reconstruction efficiency for simulated t$\bar{\text{t}}$ events with an average of 50 superimposed pileup collisions. The performance of the Patatrack reconstruction when producing pixel tracks starting from *n*-tuplets with $n_{\text{hits}}\geq 3$ and $n_{\text{hits}}\geq 4$ are represented respectively by blue squares and red circles. The performance of CMS-2018 is represented by black triangles.

The efficiency of quadruplets is sensibly improved by the Patatrack quadruplets workflow with respect to CMS-2018, as shown in Figure 7. The main reasons for this improvement are the possibility to skip a layer outside geometrical acceptance when building doublets and the usage of different doublets compatibility cuts for the barrel and the end-caps. The efficiency can be further improved including the pixel tracks built from triplets (Patatrack triplets).

**FIGURE 8:**
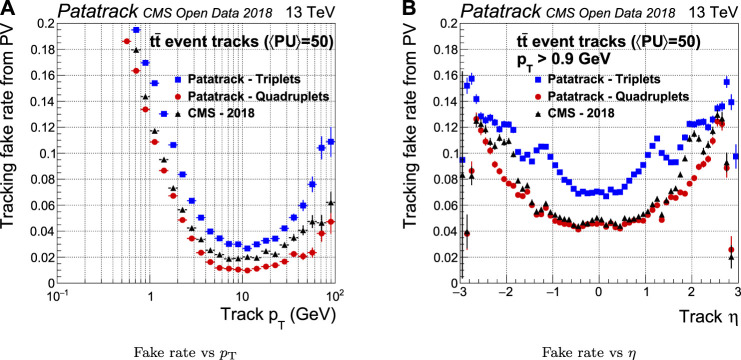
Pixel tracks reconstruction fake rate for simulated t$\bar{\text{t}}$ events with an average of 50 superimposed pileup collisions. The performance of the Patatrack reconstruction when producing pixel tracks starting from *n*-tuplets with $n_{\text{hits}}\geq 3$ and $n_{\text{hits}}\geq 4$ are represented respectively by blue squares and red circles. The performance of CMS-2018 is represented by black triangles.

The fake rate is defined as the fraction of all the reconstructed tracks coming from a reconstructed primary vertex that are not associated uniquely to a simulated track. In the case of a fake track, the set of hits used to reconstruct the track does not belong to the same simulated track. As shown in Figure 8, the fake rate performance of Patatrack quadruplets is improved with respect to the CMS-2018 pixel reconstruction in the end-cap region, mainly thanks to the different treatment of the end-caps in the Cellular Automaton. The inclusion of the pixel tracks built from Patatrack triplets slightly increases the fake rate in the tracks coming from the primary vertex, given that loosening the requirement on the number of hits decreases the quality of the selection cuts.

**FIGURE 9:**
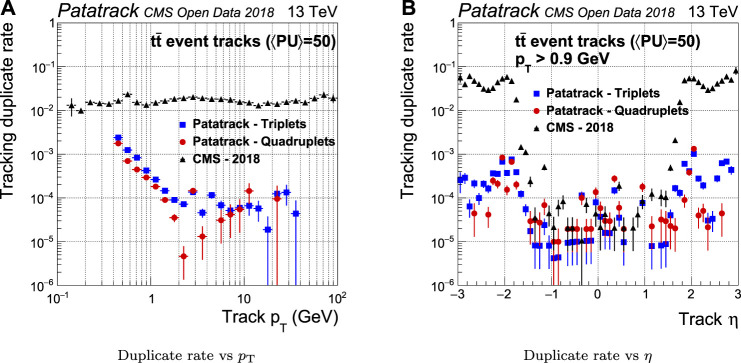
Pixel tracks reconstruction duplicate rate for simulated t$\bar{\text{t}}$ events with an average of 50 superimposed pileup collisions. The performance of the Patatrack reconstruction when producing pixel tracks starting from *n*-tuplets with $n_{\text{hits}}\geq 3$ and $n_{\text{hits}}\geq 4$ are represented respectively by blue squares and red circles. The performance of CMS-2018 is represented by black triangles.

If one simulated track is matched to more than one reconstructed tracks, the latter are defined as “duplicate.” The introduction of the *Fishbone* algorithm improves the duplicate rejection in the Patatrack workflows by up to two orders of magnitude with respect to the CMS-2018 pixel track reconstruction, as shown in Figure 9.

For historical reasons the CMS-2018 pixel reconstruction does not perform a fit on the *n*-tuplets in the transverse plane, and considers instead only the first three hits for the track parameters estimation. Furthermore, the errors on the track parameters are taken from a look-up table parameterized in η and $p_{\text{T}}$. The improvement brought in by the Broken Line fit to the accuracy of the fits can be quantified by looking at the resolution, defined as the standard deviation of the difference between the fitted and true value.

**FIGURE 10:**
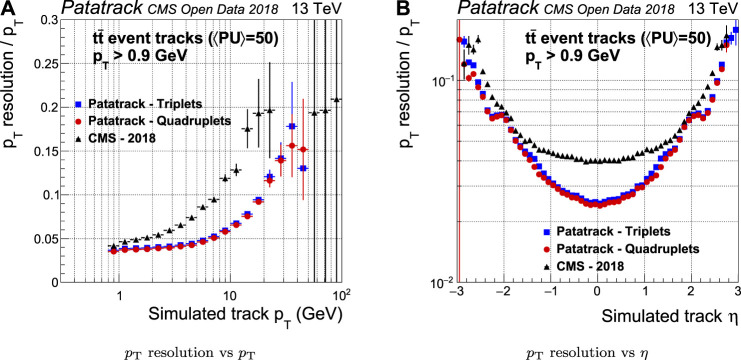
Pixel tracks $p_{\text{T}}$ resolution for simulated t$\bar{\text{t}}$ events with an average of 50 superimposed pileup collisions. The performance of the Patatrack reconstruction when producing pixel tracks starting from *n*-tuplets with $n_{\text{hits}}\geq 3$ and $n_{\text{hits}}\geq 4$ are represented respectively by blue squares and red circles. The performance of CMS-2018 is represented by black triangles.

**FIGURE 11:**
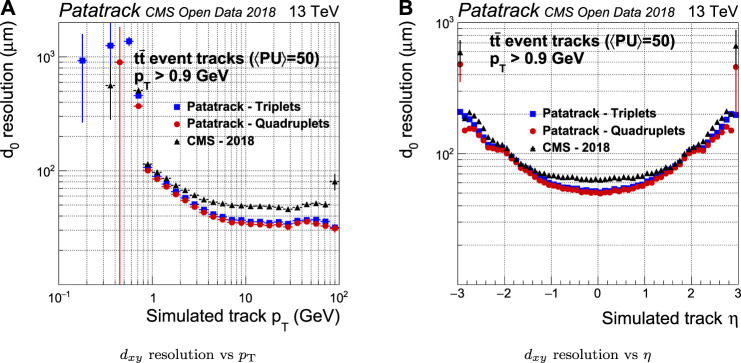
Pixel tracks transverse impact parameter resolution for simulated t$\bar{\text{t}}$ events with an average of 50 superimposed pileup collisions. The performance of the Patatrack reconstruction when producing pixel tracks starting from *n*-tuplets with $n_{\text{hits}}\geq 3$ and $n_{\text{hits}}\geq 4$ are represented respectively by blue squares and red circles. The performance of CMS-2018 is represented by black triangles.

The resolution of the estimation of the $p_{\text{T}}$ is improved by up to a factor 2 when compared to the CMS-2018 pixel tracking (Figure 10). The resolution of the transverse impact parameter $d_{0}$ improves, especially in the barrel (Figure 11).

**FIGURE 12:**
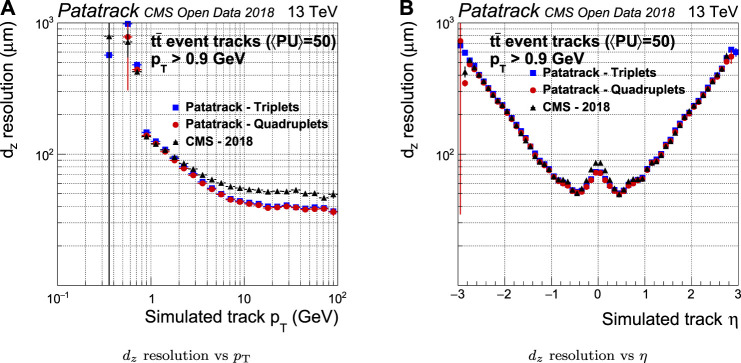
Pixel tracks longitudinal impact parameter resolution for simulated t$\bar{\text{t}}$ events with an average of 50 superimposed pileup collisions. The performance of the Patatrack reconstruction when producing pixel tracks starting from *n*-tuplets with $n_{\text{hits}}\geq 3$ and $n_{\text{hits}}\geq 4$ are represented respectively by blue squares and red circles. The performance of CMS-2018 is represented by black triangles.

The CMS-2018 pixel tracking behaves better in the longitudinal plane than it does in the transverse plane. However, the Broken Line fit’s improvement in the estimate of the longitudinal impact parameter $d_{z}$ is visible for tracks with $p_{\text{T}}>3$ GeV, as shown by Figure 12.

The number of reconstructed vertices together with the capability to separate two close-by vertices have been measured to estimate the performance of the vertexing algorithm. This capability can be quantified by measuring the vertex merge rate, i.e. the probability of reconstructing two different simulated vertices as a single one.

**FIGURE 13:**
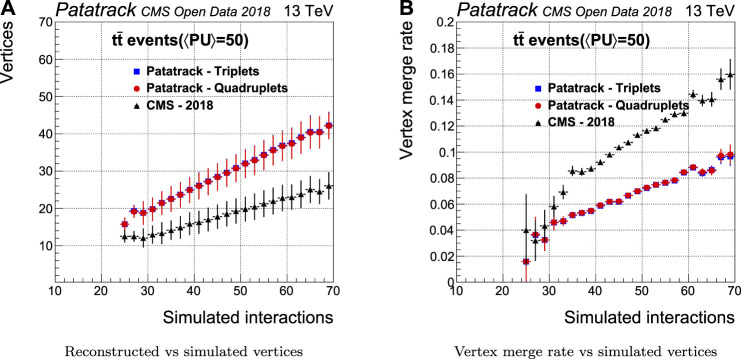
Pixel vertices reconstruction efficiency and merge rate for simulated t$\bar{\text{t}}$ events with an average of 50 superimposed pileup collisions. The performance of the Patatrack reconstruction when producing pixel tracks starting from *n*-tuplets with $n_{\text{hits}}\geq 3$ and $n_{\text{hits}}\geq 4$ are represented respectively by blue squares and red circles. The performance of CMS-2018 is represented by black triangles.

Figure 13 shows how the vertexing performance evolves with the number of simulated proton interactions. Compared to CMS-2018, the Patatrack reconstruction achieves both a higher vertex reconstruction efficiency and a lower vertex merge rate.

### 4.3. Computing Performance

The hardware and software configurations used to carry out the computing performance measurements are:

- dual socket Xeon Gold 6130 (Intel Corp, 2020), 2×16 physical cores, 64 hardware threads,
- a single NVIDIA T4 (NVIDIA Corp, 2020a),
- NVIDIA CUDA 11 with Multi-Process Service (NVIDIA Corp, 2020b),
- CMSSW 11_1_2_Patatrack (CMS Patatrack, 2020).

A CMSSW reconstruction sequence that runs only the pixel reconstruction modules as described in Section 3 was created. More than one event can be executed in parallel by different CPU threads. These can perform asynchronous operations like kernels and memory transfers, in parallel on the same GPU. The maximum amount of events that can be processed in parallel today is about 80, and is limited by the amount of allocated memory on the GPU required for each event.

In a data streaming application the measurement of the throughput, i.e. the number of reconstructed events per unit time, is a more representative metric than the measurement of the latency. The benchmark runs eight independent CMSSW jobs, each reconstructing eight events in parallel with eight CPU threads. The throughput of the CMS-2018 reconstruction has been compared to the Patatrack quadruplets and triplets workflows. The test includes the GPU and the CPU versions of the Patatrack workflows. The Patatrack workflows run with three different configurations:

1. *no copy*: the SoA containing the results stays in the memory where they have been produced;
2. *copy, no conversion*: the SoA containing the results is copied to the host, if initially produced by the GPU;
3. *copy, conversion*: the SoA containing the results is copied to the host and converted to the legacy CMS-2018 pixel tracks and vertices data formats.

These configurations are useful to understand the impact of optimizing a potential consumer of the GPU results so that it runs on GPUs in the same reconstruction sequence or so that it can consume GPU-friendly data structures, with respect to interfacing the Patatrack workflows to the existing framework without any further optimization.

The results of the benchmark are shown in Table 4. The benchmark shows that a single NVIDIA T4 can achieve almost three times the performance of a full dual socket Intel Xeon Skylake node when running the Patatrack pixel quadruplets reconstruction. Producing even better physics performance by producing also pixel tracks from triplets has the effect of almost halving the throughput. Copying the results from the GPU memory to the host memory has a small impact to the throughput, thanks to the possibility of hiding latency by overlapping the execution of kernels with copies. Converting the SoA results to the legacy data format has a small impact on the throughput as well, but comes with a hidden cost: without conversion most of the work is done by the GPU, leaving the CPU largely idle—instead, the conversion takes almost 100% of the machine’s processing power. This can be avoided by migrating all the consumers to the SoA data format.

**TABLE 4:** Throughput of the Patatrack triplets and quadruplets workflows when executed on GPU and CPU, compared to the CMS-2018 reconstruction. The benchmark is configured to reconstruct 64 events in parallel. Three different configurations have been compared: in *no copy* the result is not copied from the memory of the device where it was initially produced; in *copy, no conv.* the SoA containing the result produced on the GPU is copied to the host memory; in *conversion* the SoA containing the result is copied to the host memory (if needed) and then converted to the legacy data format used for the pixel tracks and vertices by the CMS reconstruction. The ratio to the throughput of the CMS-2018 reconstruction is indicated in parenthesis.

	Throughput in events/s				
Configuration	Triplets CPU	Triplets GPU	Quadruplets CPU	Quadruplets GPU	CMS-2018
No copy	611 (1.28)	870 (1.83)	892 (1.87)	1,386 (2.91)	476 (1.00)
Copy, no conv	—	867 (1.82)	—	1,372 (2.88)	—
Conversion	585 (1.23)	861 (1.81)	855 (1.80)	1,352 (2.84)	—

## 5. Conclusions and Future Work

The future runs of the Large Hadron Collider (LHC) at CERN will pose significant challenges on the event reconstruction software, due to the increase in both event rate and complexity. For track reconstruction algorithms, the number of combinations that have to be tested does not scale linearly with the number of simultaneous proton collisions.

The work described in this article presents innovative ways to solve the problem of tracking in a pixel detector such as the CMS one, by making use of heterogeneous computing systems in a data taking production-like environment, while being integrated in the CMS experimental software framework CMSSW. The assessment of the Patatrack reconstruction physics and timing performance demonstrated that it can improve physics performance while being significantly faster than the existing implementation. The possibility to configure the Patatrack reconstruction workflow to run on CPU or to transfer and convert results to use the CMS data format allows to run and validate the workflow on conventional machines, without any dedicated resources.

This work is setting the foundations for the development of heterogeneous algorithms in HEP both from the algorithmic and from the framework scheduling points of view. Other parts of the reconstruction, e.g. calorimeters or Particle Flow, will be able to benefit from an algorithmic and data structure redesign to be able to run efficiently on GPUs.

The ability to run on other accelerators with a performance portable code is also being explored, to ease maintainability and test-ability of a single source.

## Data Availability Statement

The source code used to perform the studies in this article can be found at https://doi.org/10.5281/zenodo.4313261. The simulated data was kindly provided by the CMS collaboration as Open Data at http://opendata.cern.ch/record/12303.

## Author Contributions

FP, MR, and VI, contributed to the development of the algorithms. AB and MK contributed to the framework development and the integration of the reconstruction in the CMS software.

## Funding

This manuscript has been partially authored by Fermi Research Alliance, LLC under Contract No. DE-AC02-07CH11359 with the U.S. Department of Energy, Office of Science, Office of High Energy Physics.

## Conflict of Interest

The authors declare that the research was conducted in the absence of any commercial or financial relationships that could be construed as a potential conflict of interest.
